# Survivin Inhibition Is Critical for Bcl-2 Inhibitor-Induced Apoptosis in Hepatocellular Carcinoma Cells

**DOI:** 10.1371/journal.pone.0021980

**Published:** 2011-08-01

**Authors:** Xiangxuan Zhao, Olorunseun O. Ogunwobi, Chen Liu

**Affiliations:** Department of Pathology, Immunology and Laboratory Medicine, University of Florida, Gainesville, Florida, United States of America; Istituto Dermopatico dell'Immacolata, Italy

## Abstract

Our study aims to study the therapeutic effects of a novel Bcl-2 inhibitor, ABT-263, on hepatocellular carcinoma (HCC) and to provide primary preclinical data for future clinical trial with ABT-263. In this study we showed that Bcl-xL and survivin were up-regulated in HCC cell lines and human liver cancer tissues. Clinic used ABT-263 single treatment had no apoptotic effects on HCC cells whereas higher doses of ABT-263 did. Interestingly, the combination treatment of ABT-263 with survivin inhibitor YM-155 could result in significant apoptosis in HCC cells. Survivin inhibition through gene silencing significantly enhanced ABT-263 to induce apoptosis in HCC cells. We found that low dose of ABT-263 single treatment resulted in ERK activation and survivin up-regulation, which might be involved in the resistance of HCC cells to ABT-263 since blockade of ERK activation sensitized ABT-263-induced apoptosis. Importantly, ABT-263 and YM-155 combination treatment had no apoptotic effects on normal human hepatocytes. Taken together, these data suggest the combination treatment of Bcl-2 inhibitor and survivin inhibition may have a great potential for liver cancer therapy.

## Introduction

Currently, there are no effective therapies for liver cancer other than surgical resection or liver transplantation in the early stages of tumor development. However, such treatments only apply to a small percentage of patients while the majorities die within 12 months of diagnosis. Therefore, new therapeutic strategies are urgently needed.

Overwhelming evidence are showing that up-regulation of the anti-apoptotic proteins of the Bcl-2 family is one of the mechanisms employed by cancer cells to evade apoptosis [1]–[3]. Small molecule inhibitors have now been designed to combat this ability of cancer cells. Of all, ABT-263 is a novel second generation orally bio-available Bcl-2 homology domain 3 (BH3)-mimetic that inhibits the anti-apoptotic Bcl-2 family proteins (Bcl-2, Bcl-xL, and Bcl-ω) [4]. It has been shown to be effective in inducing apoptosis and tumor regression in small cell lung cancer, acute lymphoblastic leukemia, multiple myeloma and lymphoid malignancies either as a single agent or in combination with other agents [5]–[8]. However, the therapeutic effects of ABT-263 on liver cancers are not known.

Meanwhile, survivin, another anti-apoptotic protein is known to be over-expressed in most cancers including liver cancers [9], [10]. Consequently, it has been becoming a very attractive target in cancer therapy. YM-155, a novel small imidazolium-based agent was shown to have specific activity against survivin with no associated systemic toxicity [11], [12]. YM-155 has demonstrated a safe profile and anti-tumor activity in a phase I trial that included patients with non-small cell lung cancer, esophageal cancer, prostate cancer cells and non-Hodgkin lymphoma [13]–[16]. Similar to ABT-263, the anti-tumor effects of YM-155 on HCC remain unknown.

In the present study, we examined the apoptotic effects of ABT-263 treatment on HCC cells. We provided evidence for the first time to show that low doses of ABT-263 treatment could not induce apoptosis in HCC cells. However, pre-incubation with survivin inhibitor YM-155 or survivin gene silencing by siRNA sensitized HCC cells to ABT-263-induced apoptosis. ERK activation and survivin up-regulation may contribute to the insensitivity of HCC cells to ABT-263. ABT-263 and YM-155 combination treatment had no apoptotic toxicity to normal human hepatocytes. Collectively, these findings provide a novel framework for understanding the mechanism of action of ABT-263 and possibly the rational integration of two agents into anti-HCC regimens.

## Materials and Methods

### Cell culture and reagents

Ethics statement: with the approval by the University of Florida Gainesville Health Science Center Institutional Review Board (IRB-01) and with informed written consent, liver cancer tissues and non-tumor liver tissues from same patients respectively were collected and analyzed. The LH86 human HCC cell line was established in our laboratory as previously described [17]. Huh7 cells were obtained from the American Type Culture Collection (ATCC) (Manassas, VA). Human hepatocellular carcinoma Huh7 and LH86 cells were grown in Dulbecco's Modified Eagle's Medium (DMEM) with 10% fetal bovine serum (Sigma, St. Louis, MO) and antibiotics (100 U/ml penicillin and 100 µg/ml streptomycin) at 37°C in 5% CO_2_. Normal primary human hepatocytes were obtained from CellzDirect Inc (Austin, TX). The cells were cultured in DMEM/F12 (1∶1) culture medium. The human normal hepatocytes used were at least 90% viable before treatment. Cells in culture were treated with ABT-263 alone or YM-155 alone (both dissolved in DMSO) or a combination of both. Untreated cells were always used as controls and exposed to equal volume of DMSO as for treated cells. ABT-263 and YM-155 were purchased from Selleck Chemical (Houston, TX); Hoechst 33258, anti-β-actin antibody, and crystal violet were obtained from Sigma (St Loius, MO); PD98059 were from Calbiochem (Gibbstown, NJ); anti-caspase 9, anti-caspase 3, anti-Bcl-xL, anti-Mcl-1, anti-survivin, anti-Bad, anti-Bax, anti-Bak, anti-ERK, and anti-p-ERK polyclonal primary antibodies were obtained from Cell Signaling Technology (Beverly, MA).

### Western blotting

Cells were grown in a monolayer in 6-well plates and treated with drugs as mentioned above until 60%-70% confluent. Cells were harvested, washed twice with 1×PBS and resuspended in lysis buffer containing Nonidet P-40 (10 mM HEPES, pH 7.4, 2 mM EGTA, 0.5% Nonidet P-40, 1 mM NaF, 1 mM NaVO_4_, 1 mM phenylmethylsulfonyl fluoride, 1 mM dithiothreitol, 50 µg/ml trypsin inhibitor, 10 µg/ml aprotinin, and leupeptin) and incubated on ice for 30 min. After centrifugation at 12,000×*g* at 4°C for 15 min, the supernatant was transferred to a new tube and the protein concentration was determined. Equivalent samples (20 µg of protein) were subjected to SDS-PAGE on 12% gels. The proteins were transferred to nitrocellulose membranes and probed with the specific primary antibodies. The Immunoreactive proteins were visualized by incubating in HRP-conjugated secondary antibodies. Chemiluminescence was detected by incubating in an equal-parts mixture of the SuperSignal West Pico stable peroxide solution and luminol/enhancer solution (Pierce, Rockford, IL) and subsequently using an image processing machine. The molecular sizes of the proteins detected were determined by comparison with prestained protein markers, Bio-Rad (Hercules, CA).

### Hoechst staining

Cells were seeded onto coverslips in 6-well plates in 10% FBS-containing medium and then treated with ABT-263 or YM-155 as mentioned above. After removal of culture medium cells were exposed to staining solution containing Hoechst 33258(1 µg/ml) at 37°C for 10–30 min. Cells with chromatin condensation were visualized and photographed using a digital fluorescence microscope (Olympus) 30 min after addition of the staining solution. Chromatin condensation is the most characteristic feature of apoptosis. Apoptotic cell death ratio was assessed by counting the number of apoptotic cells with condensed nuclei in six to eight randomly selected areas.

### Small interfering RNA (siRNA) transfection

Cells were grown in a monolayer in 6-well plates until 60%–70% confluent. SiRNA transfection was performed according to the manufacturer's instructions. Briefly, cells were plated at a density of 0.5×10^6^ cells/well in 6-well plates. Cells were transfected with 100 nM siRNA duplex mixture (Cell signaling Biotechnology, Beverly, MA) for 24 h in the presence of lipofectamine RNAiMax (Invitrogen Inc., Carlsbad, CA). A non-specific control siRNA (Cell signaling Biotechnology, Beverly, MA) was also transfected at the same concentration as the negative control.

### Colony formation assay

Colony formation assay was performed as described previously [18], [19] with modification. Cells were seeded in 6-well plates at a density of 1000 cells. Cells were untreated or treated with ABT-263, YM-155, or combination of ABT-263 and YM-155 for 48 h. After being rinsed with fresh medium, cells were allowed to grow for 14 days to form colonies, which were then stained with crystal violet (0.4 g/L; Sigma). Clonogenic assay was used to elucidate the possible differences in long-term effects the combination on human HCC cells.

### Statistical analysis

Statistical analysis was performed using Student's *t* test analysis, with *P* values<0.05 considered significant.

## Results

### Bcl-xL is up-regulated in human HCC cell lines and liver cancer tissues

**Figure 1:**
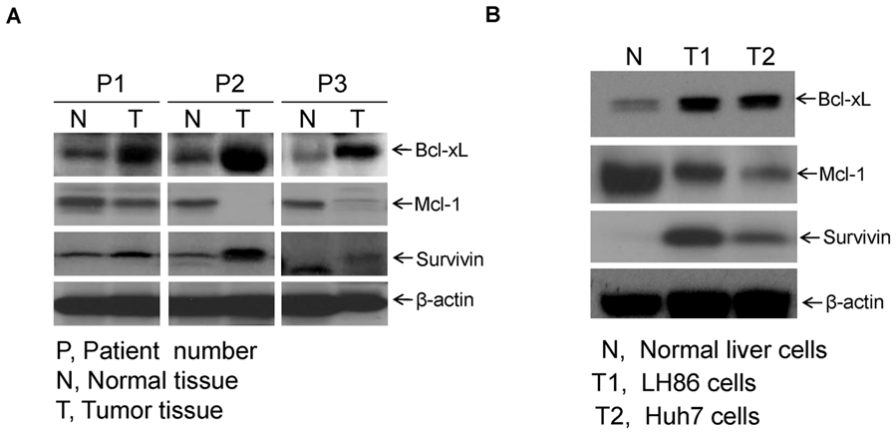
Bcl-xL is up-regulated in liver tumor tissues and HCC cells. **A**. Lysates from normal liver tissues and liver tumor tissues of different patients were prepared and subjected to Western blotting. Bcl-xL, Mcl-1, and survivin expressions were detected with specific antibodies. β-actin protein levels were assessed as an equal protein loading control (P1, P2, and P3: patient number). **B**. Cell lysates from human normal primary hepatocytes and HCC cells LH86 and Huh7 were prepared and Western blotting was performed to detect Bcl-xL, Mcl-1, and survivin expression with specific antibodies. β-actin protein levels were assessed as an equal protein loading control.

To understand the roles of Bcl-2 family of proteins in HCC, liver cancer tissues from more than 10 different patients and non-tumor liver tissues from same patients respectively were collected and analyzed for protein expression through Western blotting. As shown in Figure 1A, compared with normal liver tissues, there was consistent up-regulation of Bcl-xL and survivin protein expression in three representative liver tumor tissues, whereas Mcl-1 was down-regulated. Meanwhile, Bcl-xL, survivin and Mcl-1 protein expression was examined in normal human hepatocytes and two HCC cell lines: LH86 and Huh7. Consistent with data from the human tissue study, the two human liver cancer cell lines had increased expression of Bcl-xL and survivin in comparison to the normal primary hepatocytes. While Mcl-1 was down-regulated in both liver tumor tissues and HCC cell lines (Figure 1B). These results suggest that Bcl-2 family protein member Bcl-xL and survivin may be very important for the metastasis or development of HCC.

### HCC cells are resistant to low doses of ABT-263

**Figure 2:**
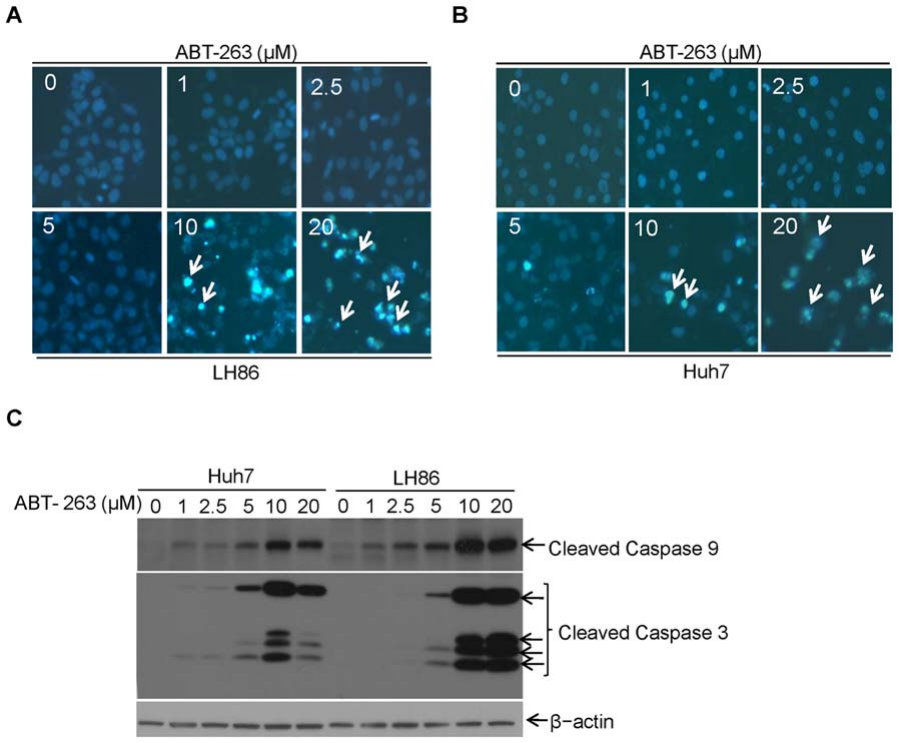
HCC cells are resistant to low doses of ABT-263. **A.** LH86 and **B.** Huh7 cells were treated with ABT-263 (0–20 µM) for up to 24 h. Apoptosis was measured through Hoechst staining to show apoptotic cells with condensed nuclei as described in ‘materials and methods’. (representative apoptotic cells were marked with white arrows in ABT-263 treatment panel). **C.** HCC cells were treated with increasing doses of ABT-263 as indicated for up to 24 h. Then cells were harvested and cell lysates were prepared and subjected to Western blotting. Caspase activation was assessed through detecting the cleaved bands of caspase 9 and caspase 3. β-actin protein levels were used as an equal protein loading control.

Given the elevated expression of Bcl-xL in HCC cells, we evaluated the therapeutic effects of ABT-263, a potent Bcl-2 family inhibitor that has been used for treatment of other cancers such as small-cell lung cancer and lymphoid malignancies clinically. A previous report has shown that ABT-263 peak and efficacy plasma concentrations were 7.7 µM [4]. Thus, in our study, cells were untreated or treated with different doses of ABT-263 (0–20 µM) for 24 h. Apoptosis assays were performed by Hoechst 33258 staining for nuclear morphology and Western blotting for caspases activation. As shown in Figure 2A and 2B, and 2C, the higher dose of ABT-263 (10–20 µM) treatment for 24 h induced significant DNA fragmentation and caspase 9 or 3 cleavage activation in HCC cells, whereas lower concentrations of ABT-263 (0–2.5 µM) had no apoptotic toxicity to HCC cells. These results suggest that HCC cells are relatively resistant to low doses of ABT-263.

### Survivin YM-155 sensitizes ABT-263-induced apoptosis in HCC cells

**Figure 3:**
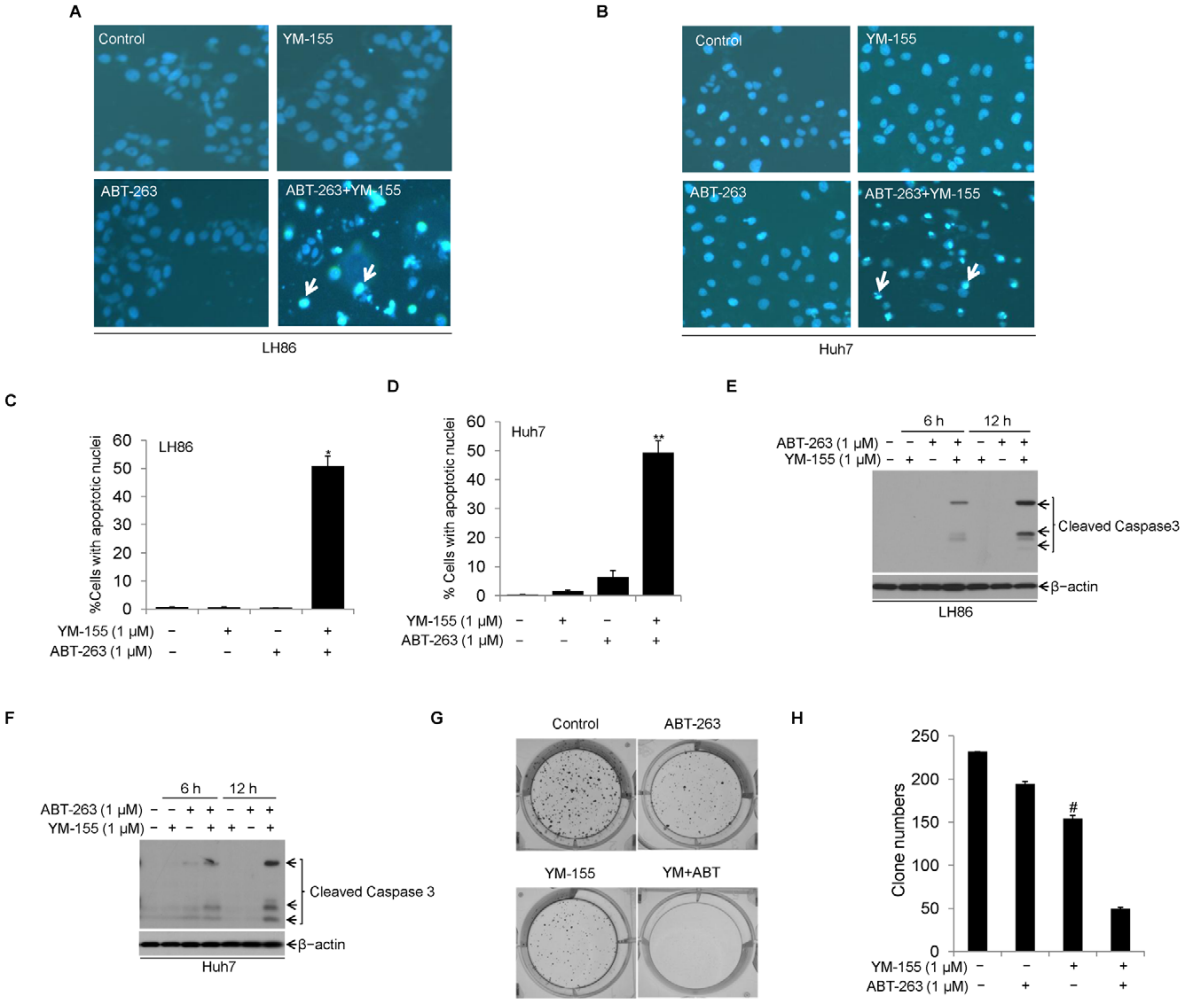
YM-155 sensitizes ABT-263-induced apoptosis in HCC cells. **A.** LH86 and **B.** Huh7 cells were untreated or treated with ABT-263(1 µM), YM-155(1 µM) or combination of ABT-263(1 µM) and YM-155(1 µM) for up to 6 h. Then apoptotic cells were assessed as in Figure 2A and 2B (representative apoptotic cells were marked with white arrows). **C.** LH86 and **D.** Huh7 cells were untreated or treated with ABT-263(1 µM), YM-155(1 µM) or combination of ABT-263(1 µM) and YM-155(1 µM) for 6 h. Cells with apoptotic nuclei were counted to determine cell death ratio (*p<0.05, **p<0.05). **E.** LH86 cells and **F.** Huh7 cells were treated as indicated and cell lysates were prepared and subjected to Western blotting. Apoptosis was evaluated through caspase 3 activation. β-actin was used as an equal protein loading control. **G.** LH86 cells grown in six-well plate were untreated (control) or treated with different conditions as indicated for 48 h. After rinsed with fresh culture medium for 3 times, cells were cultured for another two weeks. Cell colony formation assays were performed with crystal violet staining. **H.** colony number were counted to show combination treatment with ABT-263 and YM-155 resulted in reduction of clonogenesis (#p<0.05).

Survivin has been reported to be highly expressed in liver cancer cells, which contributes to the multi-drug resistance and tumor recurrences [20]–[23]. In this study, we also observed that survivin over-expressed in HCC. To find out the mechanism underlining the relative resistance of HCC cells to low dose ABT-263, we examined whether survivin is involved in the resistance of HCC cells to Bcl-2 inhibitor. Both LH86 and Huh7 cells were treated with ABT-263 (1 µM), YM-155 (1 µM), or pre-treated with YM-155 (1 µM) for 1 h followed by ABT-263 (1 µM). As shown in Figure 3A, 3B, 3C, 3D, 3E and 3F, neither ABT-263(1 µM) nor YM-155(1 µM) single treatment was able to induce apoptotic events in HCC cells. Surprisingly, a combination treatment of ABT-263 (1 µM) with YM-155 (1 µM) induced dramatic apoptosis within 6 h. Additional experiments were done to determine the anti-tumor activities of combination of ABT-263 and YM-155 inhibition when analyzed by in vitro colony formation assays. As shown in Figure 3G and 3F, median dose of ABT-263 and YM-155 combination treatment significantly reduced HCC cell proliferation. These results suggest that survivin inhibitor YM-155 can sensitize ABT-263 to induce apoptosis in HCC cells.

### Survivin down-regulation sensitizes ABT-263 to induce apoptosis in HCC cells

**Figure 4:**
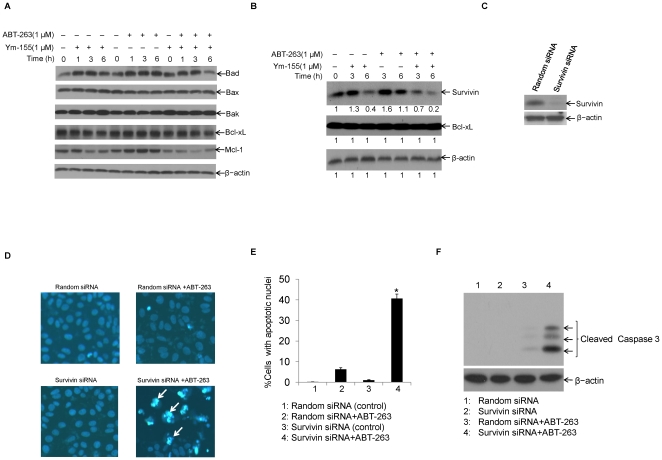
Survivin down-regulation sensitizes ABT-263-induced apoptosis in HCC cells. **A.** LH86 cells were treated as indicated and cell lysates were prepared for Western blotting. Pro-apoptotic proteins: Bax, Bad, and Bak and anti-apoptotic proteins Bcl-xL and Mcl-1 were assessed with specific antibodies respectively. β-actin was detected and served as an equal protein loading control. **B.** LH86 cells were untreated or treated with ABT-263 (1 µM), YM-155 (1 µM) or combination of ABT-263 (1 µM) and YM155 (1 µM) for up to 6 h as indicated. Then cells were harvested and cell lysates were prepared for Western blotting. Anti-survivin and anti-Bcl-xL polyclonal antibodies were used to assess protein levels for survivin and Bcl-xL respectively. β-actin was used as an equal protein loading control. The band intensities of survivin, Bcl-xL, and β-actin was qualified with Image J software. **C.** LH86 cells were transiently transfected with synthesized random siRNA (control) or survivin specific siRNA duplexes, and 48 h post-transfection, cells were subjected to Western blotting analysis with anti-survivin polyclonal antibody. β-actin was used as an equal protein loading control. **D.** LH86 cells were transfected with synthesized random control siRNA or survivin specific siRNA, and 48 h post-transfection, cells were untreated or treated with ABT-263 (1 µM) for 24 h and then subjected to Hoechst staining to show apoptotic cells with condensed nuclei (representative apoptotic cells were marked with white arrows). **E.** LH86 cells were treated as in Figure 4D and apoptosis was measured as in Figure 2A. Statistical analysis was performed for apoptosis ratio by counting the number of cells with apoptotic nuclei (*p<0.05). **F.** LH86 cells treated as in Figure 4D were harvested and cell lysates were prepared and subjected to Western blotting. Apoptosis was determined through caspase 3 activation. β-actin was used as an equal protein loading control.

YM-155 has been reported be able to inhibit survivin expression at transcription level [24]. To confirm whether the apoptosis enhancing effects observed in Figure 3 are mediated by survivin inhibition on protein level, we examined the protein expressions in HCC cells treated with ABT-263 (1 µM), YM-155 (1 µM), or the combination of YM-155 (1 µM) and ABT-263 (1 µM). As shown in Figure 4A, the combination treatment of HCC cells with ABT-263 (1 µM) and YM-155 (1 µM) for up to 6 h has no effects on the expressions of either anti-apoptotic protein Bcl-xL or pro-apoptotic proteins including Bad, Bak, and Bax. However, as expected, the presence of YM-155 significantly decreased survivin protein expression (Figure 4B, left third lane). Co-treatment of cells with ABT-263 (1 µM) and YM-155 (1 µM) induced an even greater decrease in survivin protein expression (Figure 4B, right two lanes) than that of YM-155 itself did. However, we indeed observed that ABT-263 single treatment for 3 h resulted in survivin increase (Figure 4B). To further determine survivin inhibition plays a critical role in sensitizing ABT-263 to induce apoptosis in HCC cells, we down-regulated survivin expression in HCC cells by siRNA duplexes targeted against human survivin mRNA, and then examined the expression of survivin by Western blotting (Figure 4C) and apoptotic events after ABT-263 treatments. The results demonstrated that ABT-263 induced significant apoptosis in the survivin siRNA-transfected cells, but not in siRNA Random-transfected (control) cells (Figure 4D, 4E, and 4F). In addition, Mcl-1 was also down-regulated in cells treated with YM-155 or the combination of ABT-263 and YM-155 (Figure 4A). To explore whether Mcl-1 decrease contributed to regulate YM-155 and ABT-263 co-treatment-induced apoptosis, we over-expressed Mcl-1 in HCC cells and found it could not neutralize the combination triggered cytotoxicity (data not shown). Taken together, these results suggest that survivin down-regulation plays a critical role for anti-cancer drug Bcl-2 inhibitor to induce apoptosis in HCC cells.

### Bcl-xL knocking down enhances survivin inhibitor YM-155-induced apoptotic toxicity in HCC cells

**Figure 5:**
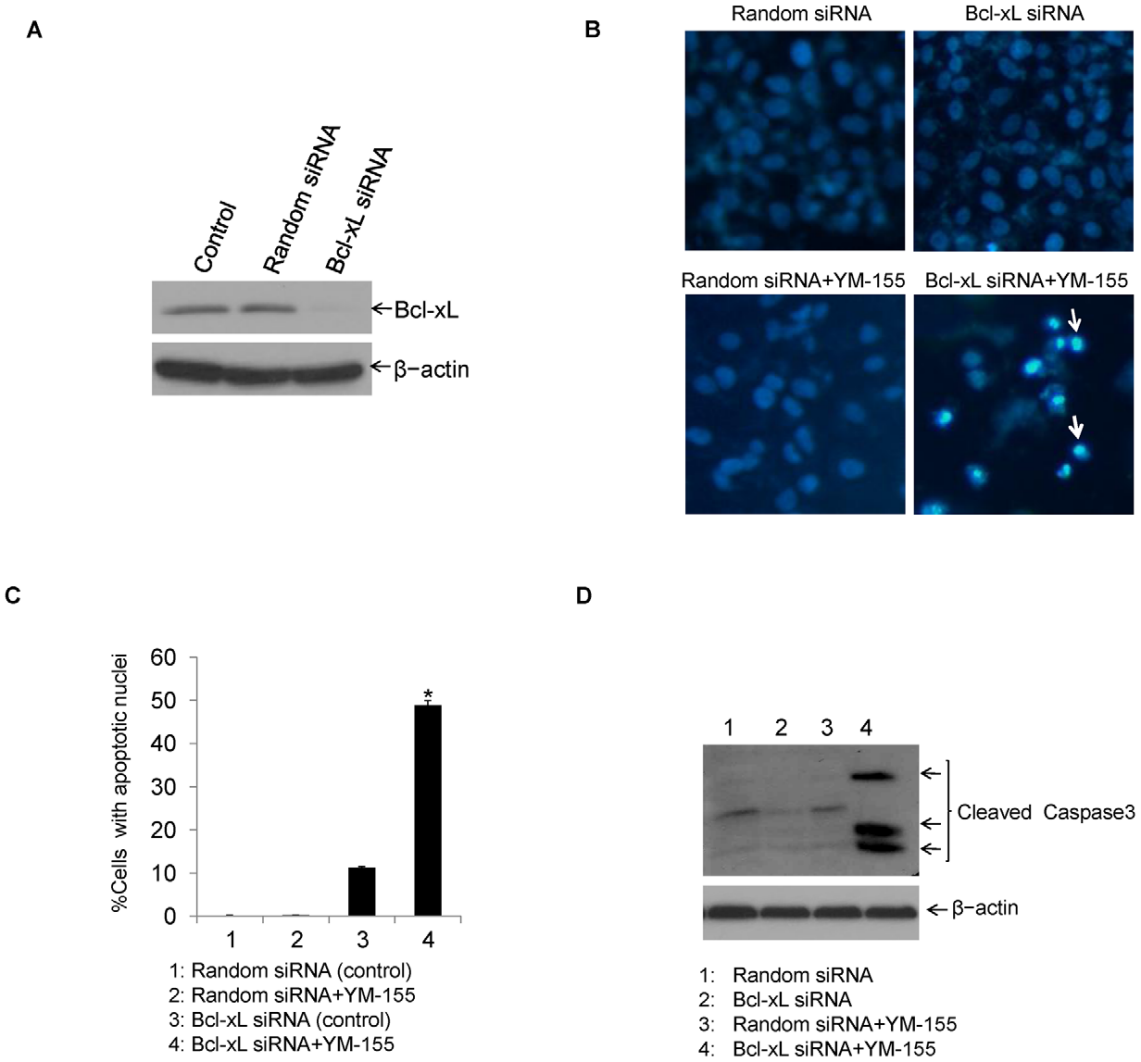
Bcl-xL down-regulation enhances YM-155-induced apoptotic toxicity in HCC cells. **A.** Huh7 cells were transiently transfected with synthesized random siRNA (control) or Bcl-xL specific siRNA duplexes, and 48 h post-transfection, cells were subjected to Western blotting analysis with anti-Bcl-xL polyclonal antibody. β-actin was used as an equal protein loading control. **B.** Huh7 cells were transfected with synthesized random control siRNA or Bcl-xL specific siRNA, and 48 h post-transfection, cells were untreated or treated with YM-155 (1 µM) for 24 h and then subjected to Hoechst staining to show apoptotic cells with condensed nuclei (representative apoptotic cells were marked with white arrows). **C.** Huh7 cells were treated as in with YM-155 as indicated and apoptosis was measured as in Figure 2A. Statistical analysis was performed for apoptosis ratio by counting the number of cells with apoptotic nuclei (*p<0.05). **D.** Huh7 cells treated were harvested and cell lyates were prepared and subjected to Western blotting. Apoptosis was determined through caspase 3 activation. β-actin was used as an equal protein loading control.

To further determine survivin inhibition can reverse the insensitivity of HCC cells in the presence of Bcl-xL activity blockade. We down-regulated Bcl-xL protein expression in HCC cells by siRNA duplexes targeted against human Bcl-xL mRNA and then treated cells with YM-155. Knock down effects of Bcl-xL were examined through Western blotting and apoptosis was assessed through nuclear staining and caspase activation. As shown in Figure 5A, compared with Random siRNA (negative control), Bcl-xL protein expression was dramatically reduced. Moreover, apoptotic data demonstrated that YM-155 induced significant DNA fragmentation and caspase 3 cleavages in the Bcl-xL siRNA-transfected cells, but not in siRNA control-transfected cells (Figure 5B, 5C, and 5D). These results suggest that survivin inhibition and Bcl-xL down-regulation is sufficient to induce apoptosis in HCC cells.

### ABT-263 induces ERK activation and survivin up-regulation in HCC cells

**Figure 6:**
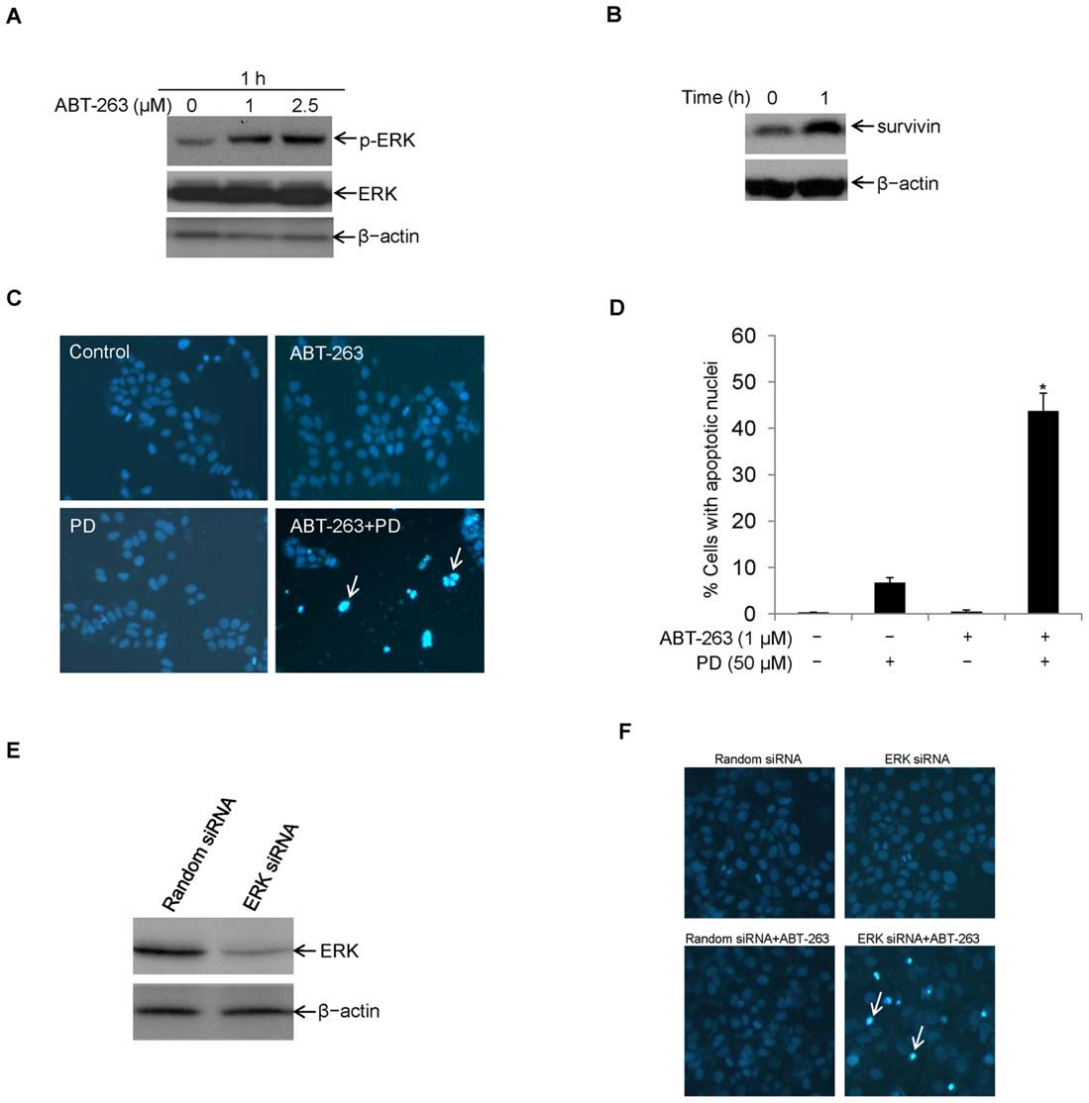
ABT-263 induces activation of ERK and survivin up-regulation in HCC cells. **A.** LH86 cells were treated with different doses of ABT-263 as indicated for 1 h. Cells were harvested and cell lysates were prepared for Western blotting. Phospho-ERK and ERK protein levels were examined with specific antibodies. β-actin was assessed and served as an equal protein loading control. **B**, LH86 cells were untreated or treated with ABT-263 (1 µM) for 1 h. Cells were harvested and cell lysates were prepared for Western blotting. Survivin expression level was examined with specific antibodies. β-actin was assessed and served as an equal protein loading control. **C.** LH86 cells were not treated (control) or treated with ABT-263(1 µM), ERK specific inhibitor PD98059 (50 µM), or pre-treated with PD98059 (50 µM) for 1 h followed by ABT-263 (1 µM) for 24 h. Apoptosis was determined by Hoechst staining to show cells with apoptotic nuclei (representative apoptotic cells were labeled with white arrows; PD: PD98059). **D.** LH86 cells were treated as in Figure 6C, apoptosis was assessed by nuclear staining and cells with apoptotic nuclei were counted as described in ‘materials and methods’ (*p<0.05). **E.** LH86 cells were untransfected or transiently transfected with synthesized random siRNA (control) or ERK specific siRNA duplexes, and 48 h post-transfection, cells were subjected to Western blotting analysis with anti-ERK polyclonal antibody. β-actin was used as an equal protein loading control. **F.** LH86 cells were transfected with synthesized random control siRNA or ERK specific siRNA, and 48 h post-transfection, cells were untreated or treated with ABT-263 (1 µM) for 24 h and then subjected to Hoechst staining to show apoptotic cells with condensed nuclei (representative apoptotic cells were marked with white arrows).

Dys-regulation and activation of Raf-MEK-ERK-survivin has been shown associated with anti-apoptotic capability of cancer cells [25]–[28]. Consequently, in order to understand why lower dose ABT-263 could not induce apoptosis of HCC cells, we examined the effects of ABT-263 on ERK-survivin signal pathway activation in HCC cells. On one hand, our data indicated that treatment of cells with ABT-263 (1–2.5 µM) for 1 h could result in the increase of phosphorylated ERK (p-ERK) but not ERK (Figure 6A). On the other hand, as shown in Figure 6B or Figure 4B, ABT-263 (1 µM) administration could result in survivin expression increase. Thus, in an attempt to know if ERK-survivin activation could protect cells against ABT-263 toxicity, cells were untreated or treated with ABT-263 (1 µM), PD98059 (50 µM), or pre-treated with PD98059 (50 µM) followed by ABT-263 (1 µM). As shown in Figure 6C and 6D, blocking ERK activation with specific inhibitor PD98059 enhanced ABT-263-indcued apoptosis in HCC cells. To further determine ERK anti-apoptotic effects on ABT-263 treated HCC cells, we knocked down ERK expression through siRNA mediated gene silencing (Figure 6E) and then administrated ABT-263. Similar results revealed that ERK depletion sensitized ABT-263-induced apoptosis (Figure 6F). These results suggest the activation of ERK-survivin may render cells to be resistant to low dose ABT-263-induced apoptosis.

### ABT-263 has no apoptotic toxicity to normal human hepatocytes

**Figure 7:**
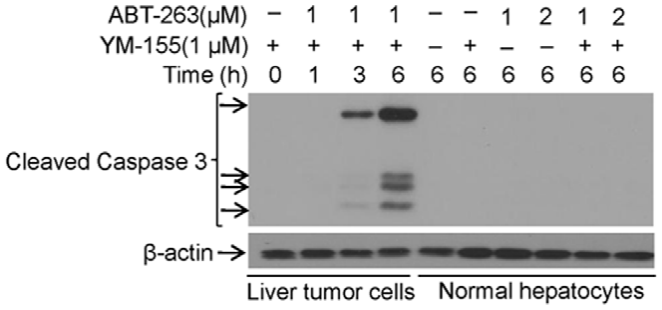
ABT-263 has no apoptotic toxicity to normal human hepatocytes. **A.** Freshly prepared normal human hepatocytes were grown in 6-well plates. After two days later, cells were treated with various conditions as indicated. Apoptosis were assessed through Western blotting to detect activation of caspase 3 with specific antibody. Cell lysates from hepatoblastoma cells (HB01) treated with a combination of ABT-263 (1 µM) and YM-155 (1 µM) for up to 6 h were set up as a positive control. β-actin was detected and served as an equal protein loading control.

Finally, we asked the question whether ABT-263, YM-155, or the combination treatment of ABT-263 and YM-155 is toxic to normal human hepatocytes *in vitro*. As shown in Figure 7, ABT-263, YM-155 single treatment, or the combination treatment of ABT-263 and YM-155 had no apoptotic toxicity in normal human hepatocytes, whereas, in positive control, the combination treatment induced significant apoptosis in liver cancer cells. These results suggest that combination treatment of Bcl-2 and survivin inhibitors is safe to normal human liver cells.

## Discussion

In the present study, we first provide evidence to show that relative low doses of ABT-263 could not promote apoptosis in human HCC cell lines. Significant apoptotic effects were observed when ABT-263 was used along with YM-155. Survivin down-regulation appears to be critical to sensitize ABT-263-induced apoptosis in HCC cells. ERK activation and survivin up-regulation induced by Low dose of ABT-263 may protect HCC cells against apoptosis. This combination treatment of ABT-263 and YM-155 has no apoptotic toxicity to normal human hepatocytes.

It is well known that Bcl-2 family members are anti-apoptotic proteins and play critical roles in tumor genesis. Hence, it is possible to induce apoptosis by targeting them with inhibitors such as ABT-263. This strategy has been shown to be promising in treatment of a number of cancers such as small cell lung cancer, acute lymphoblastic leukemia, multiple myeloma, and lymphoid malignancies [29]–[32]. However, it was unknown whether the use of ABT-263 may be an effective approach in HCC treatment. In our study, we firstly showed that Bcl-2 family protein Bcl-xL is down-regulated in normal differentiated tissues or normal human hepatocytes whereas is over-expressed in the majority of malignancies. This is consistent with previous reports that Bcl-2 family proteins were elevated in liver tumor cells [33]–[37]. In addition, unlike Bcl-xL, another Bcl-2 family anti-apoptotic protein, Mcl-1, is decreased in tumor tissues and cell lines in comparison with that in normal liver tissues and cells, of which underlining reason and function remains further study; Bcl-2 protein expression is undetectable in either liver tumor or normal samples, which is in agreement with reports that Bcl-2 is rarely expressed in liver tumor cells [38], [39]. Secondly, we did observe that very high doses of ABT-263 could induce apoptosis in HCC cells. However, there are concerns about the side effects of many chemotherapeutic agents including those in clinical use. Thus, lower doses of ABT-263-induced apoptosis were examined in HCC cells. Unfortunately, we found that HCC cells were resistant to low concentration of ABT-263. Our results are not in agreement with the studies, which suggest that low doses of ABT-263 were sufficient to induce apoptosis in cancer cells including small-cell lung cancer and lymphoma cell lines [5], [8]. This implies a possibility that in some types of cancer cells, monotherapy via inhibition of anti-apoptotic Bcl-2 family protein activity is not sufficient to trigger apoptosis. Unfortunately, insofar, there is no report to show the reason why a number of cancer cells can not be killed by Bcl-2 inhibitor. In our study, with an attempt to understand the insensitivity of HCC cells to low dose of ABT-263-induced apoptosis, we found that another anti-apoptotic protein survivin is up-regulated in liver tumor tissues or cell lines, but rarely expressed in most normal tissues and undetectable in normal liver cells, suggesting that it may facilitate cell resistant to low doses of ABT-263 treatment. Our data showed that in combination with the survivin inhibitor YM-155, very low dose of ABT-263 induced significant apoptosis in HCC cells only in 6 h. Moreover, we found that knock down of Bcl-xL significantly enhanced YM-155-induced apoptotic toxicity, which further supported Bcl-xL inhibition-induced apoptosis needs survivin inhibition. Our findings are in agreement with studies that reported that YM-155 could enhance radiation therapeutic agents-induced apoptosis in tumor cells [40]. Meanwhile, we found that the low dose of neither of these agents was able to induce apoptotic cell death of HCC cells. Third, we found that the sub-lethal dose of ABT-263 and YM-155 that combined to effectively induce apoptotic cell death in the HCC cells was not cytotoxic to human normal primary hepatocytes. Collectively, these data are very interesting and encouraging because they show that low doses of both ABT-263 and YM-155 may be used to effectively treat HCC with no associated hepatotoxicity.

We are interested in determining the mechanisms of action underlying the observed effects noted above. On one hand, our experiments showed that ABT-263, YM-155, or ABT-263 and YM-155 combination treatment had no effects on expression of pro-apoptotic proteins such as Bad, Bax, and Bak or anti-apoptotic Bcl-xL. However, YM-155 treatment could significantly induce survivin protein decrease, but not that of anti-apoptotic protein Bcl-xL, suggesting that YM-155 may sensitize ABT-263-induced apoptosis via survivin down-regulation. On the other hand, survivin knock-down through siRNA significantly enhanced low dose of ABT-263 to induce apoptosis in HCC cells, which further confirmed that survivin inactivation indeed plays critical roles to determine cell sensitivity to Bcl-2 inhibitor toxicity. Meanwhile, we observed that Mcl-1 was also decreased by either YM-155 single or combination treatment of ABT-263 and YM-155, suggesting it may contribute to sensitize ABT-263-induced apoptosis in HCC cells. However, further experiments showed that over-expression of Mcl-1 could not protect HCC cells against ABT-263 and YM-155 co-treatment- induced apoptosis. Thus, Mcl-1 appears to play no role in regulating apoptosis-induced by ABT-263 and YM-155 combination.

It has been reported that the activation of Raf-MEK-ERK-survivin signaling pathway could protect endothelial cells against gamma-radiation-induced apoptosis [41]. It is possible that incubation of low dose ABT-263 could activate ERK-survivin anti-apoptotic signal pathway to neutralize its Bcl-2 inhibition effects. In our study, we observed that low doses of ABT-263 single treatment could result in increased phosphorylation of ERK in the LH86 cells and survivin up-regulation, suggesting low dose ABT-263 can activate ERK-survivin signal pathway. These data may first provide an important and direct explanation as to why low dose ABT-263 is unable to induce apoptosis. More importantly, either using specific ERK inhibitor PD98059 to pre-treat cells or using siRNA to knock down ERK protein expression significantly sensitized sub-lethal ABT-263-induced apoptosis in HCC cells, suggesting that ERK anti-apoptotic signal pathway activation could render cells resistant to this Bcl-2 inhibitor toxicity. These findings are in agreement with previous reports that ERK-survivin signal pathway can regulate chemotherapeutic reagents-induced apoptosis [25], [26]. Finally, our results demonstrated normal human hepatocytes are resistant to the combination of ABT-263 and YM-155, of which the mechanism underlying remains to be investigated.

Overall, the data from this study have led to the following conclusions: low doses of ABT-263 alone could not induce apoptosis in HCC cells. Survivin inhibition sensitized this bcl-2 inhibitor to induce apoptosis in HCC cells, but not in normal human primary hepatocytes. ERK signal pathway activation may be involved in the protection of cells against apoptosis exerted by Bcl-2 inhibitor. Since ABT-263 and YM-155 have already shown promising results in clinical trials in other types of cancers, the current data may provide a basic clue for clinical trial of their combination in HCC treatment.

## References

[1] Danial NN, Korsmeyer SJ (2004). Cell death: critical control points.. Cell.

[2] Fesik SW (2005). Promoting apoptosis as a strategy for cancer drug discovery.. Nat Rev Cancer.

[3] Hanahan D, Weinberg RA (2000). The hallmarks of cancer.. Cell.

[4] Tse C, Shoemaker AR, Adickes J, Anderson MG, Chen J (2008). ABT-263: a potent and orally bioavailable Bcl-2 family inhibitor.. Cancer Res.

[5] Tahir SK, Wass J, Joseph MK, Devanarayan V, Hessler P (2010). Identification of expression signatures predictive of sensitivity to the Bcl-2 family member inhibitor ABT-263 in small cell lung carcinoma and leukemia/lymphoma cell lines.. Mol Cancer Ther.

[6] Ackler S, Mitten MJ, Foster K, Oleksijew A, Refici M (2010). The Bcl-2 inhibitor ABT-263 enhances the response of multiple chemotherapeutic regimens in hematologic tumors in vivo.. Cancer Chemother Pharmacol.

[7] Ackler S, Xiao Y, Mitten MJ, Foster K, Oleksijew A (2008). ABT-263 and rapamycin act cooperatively to kill lymphoma cells in vitro and in vivo.. Mol Cancer Ther.

[8] Shoemaker AR, Mitten MJ, Adickes J, Ackler S, Refici M (2008). Activity of the Bcl-2 family inhibitor ABT-263 in a panel of small cell lung cancer xenograft models.. Clin Cancer Res.

[9] Peroukides S, Bravou V, Alexopoulos A, Varakis J, Kalofonos H (2010). Survivin overexpression in HCC and liver cirrhosis differentially correlates with p-STAT3 and E-cadherin.. Histol Histopathol.

[10] Montorsi M, Maggioni M, Falleni M, Pellegrini C, Donadon M (2007). Survivin gene expression in chronic liver disease and hepatocellular carcinoma.. Hepatogastroenterology.

[11] Tolcher AW, Mita A, Lewis LD, Garrett CR, Till E (2008). Phase I and pharmacokinetic study of YM155, a small-molecule inhibitor of survivin.. J Clin Oncol.

[12] Kita A, Nakahara T, Takeuchi M, Kinoyama I, Yamanaka K (2010). [Survivin supressant: a promising target for cancer therapy and pharmacological profiles of YM155].. Nippon Yakurigaku Zasshi.

[13] Kita A, Nakahara T, Yamanaka K, Nakano K, Nakata M (2011). Antitumor effects of YM155, a novel survivin suppressant, against human aggressive non-Hodgkin lymphoma.. Leuk Res.

[14] Wang Q, Chen Z, Diao X, Huang S (2011). Induction of autophagy-dependent apoptosis by the survivin suppressant YM155 in prostate cancer cells.. Cancer Lett.

[15] Nakahara T, Kita A, Yamanaka K, Mori M, Amino N (2010). Broad spectrum and potent antitumor activities of YM155, a novel small-molecule survivin suppressant, in a wide variety of human cancer cell lines and xenograft models..

[16] Giaccone G, Zatloukal P, Roubec J, Floor K, Musil J (2009). Multicenter phase II trial of YM155, a small-molecule suppressor of survivin, in patients with advanced, refractory, non-small-cell lung cancer.. J Clin Oncol.

[17] Zhu H, Dong H, Eksioglu E, Hemming A, Cao M (2007). Hepatitis C virus triggers apoptosis of a newly developed hepatoma cell line through antiviral defense system.. Gastroenterology.

[18] Franken NA, Rodermond HM, Stap J, Haveman J, van Bree BC (2006). Clonogenic assay of cells in vitro.. Nat Protoc.

[19] Nair HK, Rao KV, Aalinkeel R, Mahajan S, Chawda R (2004). Inhibition of prostate cancer cell colony formation by the flavonoid quercetin correlates with modulation of specific regulatory genes.. Clin Diagn Lab Immunol.

[20] Yang Y, Zhu J, Gou H, Cao D, Jiang M (2010). Clinical significance of Cox-2, Survivin and Bcl-2 expression in hepatocellular carcinoma (HCC).. Med Oncol.

[21] Mamori S, Asakura T, Ohkawa K, Tajiri H (2007). Survivin expression in early hepatocellular carcinoma and post-treatment with anti-cancer drug under hypoxic culture condition.. World J Gastroenterol.

[22] Yan G, Huang AL, Tang N, Zhang BQ, Pu D (2003). [Inhibition of survivin expression in liver cancer cells by shRNA].. Zhonghua Gan Zang Bing Za Zhi.

[23] Ito T, Shiraki K, Sugimoto K, Yamanaka T, Fujikawa K (2000). Survivin promotes cell proliferation in human hepatocellular carcinoma.. Hepatology.

[24] Nakahara T, Takeuchi M, Kinoyama I, Minematsu T, Shirasuna K (2007). YM155, a novel small-molecule survivin suppressant, induces regression of established human hormone-refractory prostate tumor xenografts.. Cancer Res.

[25] Jeong JC, Kim MS, Kim TH, Kim YK (2009). Kaempferol induces cell death through ERK and Akt-dependent down-regulation of XIAP and survivin in human glioma cells.. Neurochem Res.

[26] Li L, Gao Y, Zhang L, Zeng J, He D, Sun Y (2008). Silibinin inhibits cell growth and induces apoptosis by caspase activation, down-regulating survivin and blocking EGFR-ERK activation in renal cell carcinoma.. Cancer Lett.

[27] Siddiqa A, Long LM, Li L, Marciniak RA, Kazhdan I (2008). Expression of HER-2 in MCF-7 breast cancer cells modulates anti-apoptotic proteins Survivin and Bcl-2 via the extracellular signal-related kinase (ERK) and phosphoinositide-3 kinase (PI3K) signalling pathways.. BMC Cancer.

[28] Jalal HS, Inanami O, Hamasu T, Takahashi M, Kashiwakura I (2004). Activation of c-kit by stem cell factor induces radioresistance to apoptosis through ERK-dependent expression of survivin in HL60 cells.. J Radiat Res (Tokyo).

[29] Gandhi L, Camidge DR, Ribeiro de OM, Bonomi P, Gandara D (2011). Phase I Study of Navitoclax (ABT-263), a Novel Bcl-2 Family Inhibitor, in Patients With Small-Cell Lung Cancer and Other Solid Tumors.. J Clin Oncol. JCO.

[30] Ackler S, Mitten MJ, Foster K, Oleksijew A, Refici M (2010). The Bcl-2 inhibitor ABT-263 enhances the response of multiple chemotherapeutic regimens in hematologic tumors in vivo.. Cancer Chemother Pharmacol.

[31] Ackler S, Xiao Y, Mitten MJ, Foster K, Oleksijew A, Refici M (2008). ABT-263 and rapamycin act cooperatively to kill lymphoma cells in vitro and in vivo.. Mol Cancer Ther.

[32] Shoemaker AR, Mitten MJ, Adickes J, Ackler S, Refici M (2008). Activity of the Bcl-2 family inhibitor ABT-263 in a panel of small cell lung cancer xenograft models.. Clin Cancer Res.

[33] Guo XZ, Shao XD, Liu MP, Xu JH, Ren LN (2002). Effect of bax, bcl-2 and bcl-xL on regulating apoptosis in tissues of normal liver and hepatocellular carcinoma.. World J Gastroenterol.

[34] Garcia EJ, Lawson D, Cotsonis G, Cohen C (2002). Hepatocellular carcinoma and markers of apoptosis (bcl-2, bax, bcl-x): prognostic significance.. Appl Immunohistochem Mol Morphol.

[35] Ravazoula P, Tsamandas AC, Kardamakis D, Gogos C, Karatza C (2002). The potential role of bcl-2 mRNA and protein exression in hepatocellular carcinomas.. Anticancer Res.

[36] Feng D, Zheng H, Shen M, Cheng R, Yan Y (1999). [Regulation of p53 and bcl-2 proteins to apoptosis and cell proliferation in liver cirrhosis and hepatocellular carcinoma].. Hunan Yi Ke Da Xue Xue Bao.

[37] Fiorentino M, D'Errico A, Altimari A, Barozzi C, Grigioni WF (1999). High levels of BCL-2 messenger RNA detected by in situ hybridization in human hepatocellular and cholangiocellular carcinomas.. Diagn Mol Pathol.

[38] Takahashi M, Saito H, Okuyama T, Miyashita T, Kosuga M (1999). Overexpression of Bcl-2 protects human hepatoma cells from Fas-antibody-mediated apoptosis.. J Hepatol.

[39] Kim Y, Yoon JW, Xiao X, Dean NM, Monia BP (2007). Selective down-regulation of glioma-associated oncogene 2 inhibits the proliferation of hepatocellular carcinoma cells.. Cancer Res.

[40] Satoh T, Okamoto I, Miyazaki M, Morinaga R, Tsuya A (2009). Phase I study of YM155, a novel survivin suppressant, in patients with advanced solid tumors.. Clin Cancer Res.

[41] Kumar P, Coltas IK, Kumar B, Chepeha DB, Bradford CR (2007). Bcl-2 protects endothelial cells against gamma-radiation via a Raf-MEK-ERK-survivin signaling pathway that is independent of cytochrome c release.. Cancer Res.

